# Effect of Subthalamic Stimulation on Voice and Speech in Parkinson’s Disease: For the Better or Worse?

**DOI:** 10.3389/fneur.2013.00218

**Published:** 2014-01-13

**Authors:** Sabine Skodda, Wenke Grönheit, Uwe Schlegel, Martin Südmeyer, Alfons Schnitzler, Lars Wojtecki

**Affiliations:** ^1^Department of Neurology, Knappschaftskrankenhaus, Ruhr-University of Bochum, Bochum, Germany; ^2^Center for Movement Disorders and Neuromodulation, Institute of Clinical Neuroscience and Medical Psychology, Department of Neurology, Medical Faculty, Heinrich-Heine University, Düsseldorf, Germany

**Keywords:** deep brain stimulation of the subthalamic nucleus, Parkinson’s disease, dysarthria, quality of voice, speech impairment, perceptual analysis of speech, acoustic speech analysis

## Abstract

**Background:** Deep brain stimulation of the subthalamic nucleus, although highly effective for the treatment of motor impairment in Parkinson’s disease (PD), can induce speech deterioration in a subgroup of patients. The aim of the current study was to survey (1) if there are distinctive stimulation effects on the different parameters of voice and speech and (2) if there is a special pattern of preexisting speech abnormalities indicating a risk for further worsening under stimulation.

**Methods:** *N* = 38 patients with PD had to perform a speech test without medication with stimulation ON (StimON) and stimulation OFF (StimOFF). Speech samples were analyzed: (1) according to a four-dimensional perceptual speech score and (2) by acoustic analysis to obtain quantifiable measures of distinctive speech parameters.

**Results:** Quality of voice was ameliorated with StimON, and there were trends of increased loudness and better pitch variability. *N* = 8 patients featured a deterioration of speech with StimON, caused by worsening of articulation or/and fluency. These patients already had more severe overall speech impairment with characteristic features of articulatory slurring and articulatory acceleration under StimOFF condition.

**Conclusion:** The influence of subthalamic StimON Parkinsonian speech differs considerably between individual patients, however, there is a trend to amelioration of voice quality and prosody. Patients with stimulation-associated speech deterioration featured higher overall speech impairment and showed a distinctive pattern of articulatory abnormalities at baseline. Further investigations to confirm these preliminary findings are necessary to allow neurologists to pre-surgically estimate the individual risk of deterioration of speech under stimulation.

## Introduction

Chronic deep brain stimulation of the subthalamic nucleus (STN-DBS) has been shown to be superior over best medical treatment in patients with motor fluctuations in Parkinson’s disease (PD) (1, 2). However, the effects of STN-DBS on voice and speech have been found to be variable or even adverse, at least in a subgroup of patients. According to overall perceptual evaluation based upon the speech item of the Unified Parkinson’s Disease Rating Scale/Motor Score (UPDRS III), the prevalence of dysarthria under STN-DBS has been reported to vary between 1% after 6 months up to 70% at 3 years follow-up with an average of 9.3% (3–5). Furthermore, “communication” was the only item of the PD Questionnaire that showed deterioration under STN-DBS in the recently published EARLYSTIM study (6). However, there are also reports of an amelioration of distinctive parameters of voice, loudness, and non-speech vocal measures in individual PD patients under STN-DBS (7–13). As a possible explanation for these contradictory findings, it has been proposed that STN-DBS could reduce a few distinctive dysarthrophonic symptoms such as reduced loudness and glottic tremor in PD. However, these beneficial effects could be outweighed by a general dysarthrogenic impact on prosodic and articulatory functions leading to reduced overall speech intelligibility (7, 14–17). Furthermore, as a possible hint for a negative effect on basal motor speech performance, STN-DBS was found to induce abnormalities in the speed and regularity of non-speech syllable repetition (18). In respect to these conflicting results, there is still a lack of reliable predictability of speech motor outcome in the individual patient, although clinical and surgical factors (e.g., anatomic location of the electrode contact, amplitude of current in the right and left STN) seem to be critical for the speech outcome under STN-DBS (19).

The aim of the current study was to analyze the effect of STN-DBS on voice and speech in a group of PD patients based upon perceptual and acoustic analysis of distinctive speech modalities. It had been chosen to test patients without the additional effect of dopaminergic medication to identify the exclusive impact of STN-DBS with stimulation settings previously optimized for best overall motor performance in order to test patients under their “naturalistic” stimulation situation. According to previous studies, it had been hypothesized that there would be a differential outcome of patients’ speech performance under stimulation and therefore, it was further intended to better characterize the pattern of changes within the single speech modalities. In particular, attention was given to the expected subgroup of patients with a deterioration of speech performance under stimulation in order to identify patterns of preexisting speech impairment that might serve as “risk profile” for further worsening under STN-DBS.

## Patients and Methods

From 2008 to 2010, 38 patients with idiopathic PD and chronic bilateral STN-DBS were recruited for this study. The diagnosis of PD was based upon the UK Parkinson’s Disease Society Brain Bank Criteria (20). After an overnight wash out period of medication, each patient was tested under two conditions OFF medication: stimulation OFF (StimOFF) and stimulation ON (StimON) and underwent a neurological examination according to UPDRS Motor Scale (UPDRS III) immediately before performing the speech task. Patients’ characteristics are summarized in Table 1.

**Table 1:** **Participants’ characteristics/results of the comparison of perceptual speech analysis**.

	Control group	PD group		
		StimOFF/MedOFF	StimON/MedOFF	
	Mean/SD/range	Mean/SD/range	Mean/SD/range	
Age (y)	67.14/8.03/48–80	65.69/7.85/45–77		
Age at DBS surgery		62.13/8.01/43–73		
Disease duration (y)		15.71/6.07/6–28		
Disease duration at DBS surgery		12.24/6.97/5–24		

	**Median/1.–3. quartile**	**Median/1.–3. quartile**	**Median/1.–3. quartile**

UPDRS III		39/32.75–47	21.37/10.17/7–50	*p* < 0.0001
UPDRS III axial subscore (% of overall UPDRS score)		11/8.75–16 (*29.50*%)	7/5–10.25 (*37.74*%)	*p* < 0.0001
UPDRS III tremor subscore (% of overall UPDRS score)		3.5/0–8 (*11.95*%)	0/0–2.25 (*6.47*%)	*p* < 0.0001
UPDRS III akinesia subscore (% of overall UPDRS score)		25/19–29.50 (*60.72*%)	13/7–20.25 (*61.90*%)	*p* < 0.0001
UPDRS III speech item		1/0–2	1/0–2	n.s.
Perceptual speech score	1/0–2***	5/4–7	5/3–7	n.s. (*p* = 0.085)
Voice	0/0–1****	1/1–2	1/1–1.25	*p* = 0.001
Articulation	0/0–0****	2/1–2	2/1–2	n.s
Fluency	0/0–0****	1/1–2	1/1–2	n.s
Prosody	0/0–0****	1/0–1.25	1/0–1	n.s.

***p < 0.001.

****p < 0.0001 related to the comparison between control group and PD group with StimOFF/MedOFF.

*y, years; SD, standard deviation; n.s., not significant; UPDRS III, unified Parkinson’s disease rating scale, Part III: motor part*.

As control group we tested 30 age-matched healthy persons.

All participants were native German speakers, and the speech evaluation was based upon a German text. For the speech test, each participant had to read a given text composed of four phonetically balanced sentences; furthermore, participants had to produce the vowel, /a/, for as long as possible. Speech samples were digitally recorded using a commercial audio software (Steinberg WaveLab^®^/Steinberg Media Technologies GmbH, Hamburg, Germany) and a head-set microphone with a defined mouth to microphone distance. Speech records of the reading task were perceptually analyzed independently by two examiners (Sabine Skodda and Wenke Grönheit) who were blinded for the speakers’ condition, according to a four-dimensional scoring system that is used for the description of Parkinsonian dysarthria in our clinic (Table 2). Inter-rater reliability was high with *w* = 0.923; in cases of divergent ratings, the higher score was chosen for the further analysis.

**Table 2:** **Perceptual speech score**.

Speech modality		Definition
Voice	0	Normal
	1	Voice quality slightly hoarse, slightly reduced loudness, intermittently present
	2	Voice quality hoarse or tremulous, slightly reduced loudness, continuously present
	3	Voice quality hoarse or tremulous, markedly reduced loudness
	4	Marked reduction of voice quality, whispery, or scratchy voice
Articulation	0	Normal articulation
	1	Slightly reduced articulatory accuracy, intermittently present
	2	Slightly reduced articulatory accuracy, continuously present
	3	Markedly reduced articulatory accuracy, slightly reduced intelligibility
	4	Markedly reduced intelligibility
Tempo/fluency	0	Normal speech tempo and distribution of speech pauses
	1	Slightly reduced or accelerated speech tempo, intermittently present
	2	Rushes of speech and prolonged pauses, not very pronounced or only intermittently present; or slightly reduced speech tempo
	3	Rushes of speech and prolonged pauses, very pronounced or continuously present; or markedly reduced speech tempo
	4	Palilalia
Prosody	0	Normal pitch variability
	1	Slightly monotone
	2	Extremely monotone

Additionally, acoustic analysis of speech was performed for several speech parameters for the objective description of voice, articulation, fluency, and prosody by the use of PRAAT (21) (Table 3). Jitter, shimmer, and noise to harmonics-ratio as measures of *voice quality* were based upon the analysis of sustained phonation (22). Mean fundamental frequency (meanF_0_) of the reading task was taken as measure of *phonation*. *Loudness* was defined as average sound pressure level of the entire reading task. Description of *intonation variability* was based upon standard deviation (SD) of the fundamental frequency (F_0_SD). Analysis of *speech rate* was performed by measuring the length of each syllable and each pause respectively based on the oscillographic sound pressure signal. Besides the conventional speech rate variables as net speech rate (NSR) and pause ratio (PR%), we additionally defined the percent ratio of pauses within polysyllabic words (Pinw%), which can be taken as a measure of precision of *stop consonant articulation* (23). *Articulatory acceleration* (AA) in the course of reading was defined as the difference of NSR between the first and last sentence with values >0 indicating acceleration (23). Description of *vowel articulation* was based upon the recently established vowel articulation index/VAI, which is a surrogate parameter of the first and second formant frequencies (F1 and F2) of the three corner vowels, /α/, /i/, and /u/ (24, 25). Since meanF_0_, F_0_SD, and VAI are related to the speaker’s pitch of voice, the comparison of these parameters between PD patients and controls were performed separately for both genders.

**Table 3:** **Abbreviations and definitions of the speech parameters**.

Speech modality	Parameter	Definition
Voice	Jitter (measure of microperturbations of frequency)	Average absolute difference between consecutive differences between consecutive periods, divided by the average period
	Shimmer (measure of microperturbations of amplitude)	Average absolute difference between consecutive differences between the amplitude of consecutive periods
	Noise to harmonics ratio (nhR)	Automatic comparison of harmonic (periodically recurring) and inharmonic sound fractions
	Loudness in dB	Average sound pressure level calculated for entire reading task
	MeanF_0_	Average fundamental frequency F_0_ calculated for entire reading task
Articulation	Vowel articulation index (VAI)	Comprehensive measure of the “working space” for vowels based upon the extraction of formant frequencies of defined vowels of the reading task according to the formula VAI = (F2/i/ + F1/α/)/(F1/i/ + F1/u/ + F2/u/ + F2/α/)
	Percentage of pauses within polysyllabic words (Pinw%)	Percentage of pauses within polysyllabic words of total speech pauses (periods of silence <10 ms)
Tempo/fluency	Net speech rate (NSR)	Net production of syllables per second based upon reading task
	Pause ratio (PR%)	Percentage of pause rate based upon the reading task
	Articulatory acceleration (AA)	Difference between NSR of the first and last sentence of the reading task (values >0 display acceleration)
Prosody	F_0_SD	Standard deviation of fundamental frequencies calculated for the reading task as a measure of pitch variability

Winstat© (Bad Krotzingen/Germany) was used for statistical analyses. ANOVA and paired *t*-test were performed for the comparison of patients with the control group and intra-group comparison (StimOFF vs. StimON). The variables were normally distributed (Shapiro–Wilk test). Continuous variables are presented using mean±SD. Discrete data are reported with median and quartile deviation. For the calculation of inter-rater reliability, Kendall’s coefficient of concordance was used. Spearman rank test was used to perform correlation analyses in order to account for possible outliers especially within the subgroup analyses. Due to the exploratory nature of the study, no adjustments for multiple comparisons were made, and the level of significance was set at *p* < 0.05.

Our study was in compliance with the Helsinki Declaration and had been approved by the local Ethics Committees. Written informed consent was obtained from each participant.

## Results

### Comparison of control group with PD group StimOFF

Based upon perceptual ratings, the control group featured a significantly better performance of voice, articulation, fluency, and prosody. This was reflected in the acoustic analysis by lower values for jitter, shimmer, and noise to harmonics-ratio indicating a better voice quality, by higher sound pressure levels, higher values for the measures of articulatory precision (Pinw%, VAI) and pitch variability (F_0_SD), and an elevated meanF_0_ in female speakers. Measures of speech rate and PR% showed no significant differences between the control and the PD group in the StimOFF condition.

This pattern of speech abnormalities was in general preserved also under StimON: there were significantly worse values for shimmer, loudness, VAI, Pinw%, and F_0_SD, whereas no significant differences compared to the control group were seen concerning shimmer, meanF_0_, NSR, PR%, and AA (numerical data are given in Tables 1 and 4).

**Table 4:** **Comparison between the PD groups with stimulation OFF and ON and comparison between the PD group/StimOFF and the control group**.

	Control (*n* = 30, 15 male)	PD patients (*n* = 38, 22 male)		Comparison StimOFF/Med OFF vs. StimON/MedOFF
		StimOFF/MedOFF	StimON/MedOFF	
	Mean/SD	Mean/SD	Mean/SD
Jitter	1.247/0.704**	2.065/1.941	1.857/2.040	n.s
Shimmer	5.613/2.722***	10.733/7.246	9.272/5.980	n.s.
nhR	0.038/0.033**	0.086/0.097	0.078/0.089	n.s.
Loudness (dB)	78.92/2.10****	70.02/7.54	71.02/8.10	n.s. (*p* = 0.064)
MeanF_0_ male	118.46/13.98	117.96/19.40	121.67/18.39	n.s.
MeanF_0_ female	192.83/8.51*	169.28/42.59	191.01/23.47	n.s. (*p* = 0.05)
VAI male	0.781/0.070****	0.668/0.074	0.657/0.058	n.s.
VAI female	0.914/0.050****	0.721/0.060	0.714/0.069	n.s.
Pinw%	29.77/8.53****	15.53/10.66	14.85/10.90	n.s.
NSR	5.23/0.56	5.30/1.06	5.23/1.13	n.s.
PR%	18.61/4.17	17.29/9.06	18.31/9.53	n.s.
AA	0.22/0.34	0.35/0.57	0.35/0.57	n.s.
F_0_SD male	20.13/6.78**	14.58/4.29	15.42/4.45	n.s.
F_0_SD female	31.86/6.11****	16.76/5.31	19.69/4.90	n.s.

*p < 0.05; **p < 0.01; ***p < 0.001; ****p < 0.0001.

### Correlations between perceptual and acoustic analysis

In the PD group in the OFF condition, there were found some significant correlations between “voice” and the jitter (*r* = 0.343, *p* = 0.019) and shimmer values (*r* = 0.289, *p* = 0.041), between “articulation” and Pinw% (*r* = −0.277, *p* = 0.046), but not with VAI, between “fluency” and NSR (*r* = 0.385, *p* = 0.008) and AA (*r* = 0.478, *p* = 0.001), but not with PR%, and between “prosody” and F_0_SD (*r* = −0.311, *p* = 0.028). In general, similar correlations between perceptual and acoustic measures were also observed in the ON condition (data not shown).

In the control group, no close correlations were expected because of the low overall speech impairment with an average perceptual sum speech score of 0.88. Accordingly, there were only weak correlations between the perceptual categories “voice,” “articulation,” “fluency,” and “prosody” on the one hand, and the accordant acoustic measures on the other (“voice”/jitter: *r* = 0.417, *p* = 0.021; no significant correlations with shimmer, nhR, and loudness; “articulation”/VAI: *r* = 0.307, *p* = 0.072, no correlation with Pinw%; “fluency”/PR%: *r* = 0323, *p* = 0.062, “fluency”/AA: *r* = 0.339, *p* = 0.052, no correlation with NSR; “prosody”/F_0_SD: *r* = −0.388, *p* = 0.031).

### Comparison within the PD group: StimOFF vs. StimON: Groupwise comparisons

Total UPDRS III scores as well as the chosen UPDRS subscores (axial, tremor, akinesia) were significantly ameliorated under StimON condition, whereas UPDRS speech score (item 18) showed no significant difference. The more detailed perceptual speech score showed a tendency to reduced overall ratings that were mainly caused by an amelioration of voice quality in the StimON condition whereas the other speech modalities remained widely unchanged. Similar results were observed with the measures of the acoustic analysis where only sound pressure levels and meanF_0_ in female PD speakers showed a (non-significant) tendency to amelioration. However, no further changes were observed between StimOFF and StimON conditions in the groupwise comparison (numerical data are given in Tables 1 and 4).

### Comparisons within the PD group and characterization of the subgroup with speech deterioration

In an evaluation of the different qualitative speech modalities in the individual patients, 12/38 patients showed no difference in the sum perceptual speech score, 18/38 showed an amelioration of the sum speech score (13 patients improved by 1 point, 4 patients by 2 points, and 1 patient by 3 points respectively), which was mainly caused by an improvement of voice (*n* = 12) and less often by amelioration of articulation (*n* = 7), prosody (*n* = 4), or fluency (*n* = 1). These improvements showed no correlation with improvement of motor symptoms as tremor, akinesia, or axial symptoms based upon the accordant UPDRS III subscores.

In 8/38 patients, there was a deterioration of speech (6 patients worsened by 1 point, 1 patient each worsened by 2 points and 3 points respectively) with worsening of articulation in 4 patients, of fluency in 3, of prosody in 3, and of voice in 1 patient. The group of patients with speech deterioration showed no significant difference concerning age and disease duration, however, UPDRS III was significantly higher in StimOFF. No differences were seen with the tremor, akinesia, or axial UPDRS subscores. The UPDRS speech item showed a tendency to higher values, however, without statistical significance. In 5/8 patients with speech deterioration, the right STN was stimulated with higher current amplitudes (compared to 11/30 in the subgroup without worsening of speech) than the left-side STN due to asymmetry of motor symptoms going along with higher total electric energy delivered/TEED (26) since pulse width, frequency, and impedances (measured in *n* = 30 patients) showed no significant differences (see Table 5).

**Table 5:** **Listing and description of the stimulation parameters, impedance, and electrode settings for the entire PD group and the two subgroups with and without worsening of speech under StimON**.

	Left STN					Right STN				
	Ampl. (V)	Pulse width (μs)	Freq. (Hz)	Imp. (Ω)*	Elec. comb.	Ampl. (V)	Pulse width (μs)	Freq. (Hz)	Imp. (Ω)*	Elec. comb.
	Median/SD/ 1.–3. quartile		Median/SD/ 1.–3. quartile	Median/SD/ 1.–3. quartile		Median/SD/ 1.–3. quartile
Entire PD group/*n* = 38	3.19/0.74	*n* = 34: 60	*n* = 2: 125	1048/94.79/71–2972	*n* = 5: 0-	3.25/0.67	*n* = 35: 60	*n* = 31: 130	958/90.77/60–2190	*n* = 6: 0-
	2.8–3.7	*n* = 4: 90	*n* = 31: 130		*n* = 15: 1-	2.8–3.8	*n* = 3: 90	*n* = 2: 125		*n* = 10: 1-
			*n* = 1: 140		*n* = 15: 2-			*n* = 1: 140		*n* = 16: 2-
			*n* = 3: 185		*n* = 3: 3-			*n* = 3: 185		*n* = 6: 3-
			*n* = 1: 200					*n* = 1: 200	
Speech worsening under StimON/*n* = 8	3.53/0.70/3.0–3.7	*n* = 6: 60	*n* = 8: 130		*n* = 2: 0-	3.66/0.51/3.3–4.2	*n* = 6: 60	*n* = 8: 130		*n* = 2: 0-
		*n* = 2: 90			*n* = 4: 1-		*n* = 2: 90			*n* = 1: 1-
					*n* = 2: 2-					*n* = 2: 2-
					*n* = 0: 3-					*n* = 3: 3-
No speech worsening/*n* = 30	3.10/0.74/2.7–3.7	*n* = 28: 60	*n* = 2: 125		*n* = 3: 0-	3.14/0.67/2.6–3.7	*n* = 29: 60	*n* = 2: 125		*n* = 4: 0-
		*n* = 2: 90	*n* = 13: 130		*n* = 11: 1-		*n* = 1: 90	*n* = 13: 130		*n* = 9: 1-
			*n* = 1: 140		*n* = 13: 2-			*n* = 1: 140		*n* = 14: 2-
			*n* = 3: 185		*n* = 3: 3-			*n* = 3: 185		*n* = 3: 3-
			*n* = 1: 200					*n* = 1: 200	

Ampl., amplitude; freq., frequency; imp., impedance (note that there are only measures for *n* = 30 patients, the missing data exclusively concern the group of “no speech worsening”)* elec. comb., electrode combination.

*The amplitude of the right STN stimulation was significantly higher (*p* = 0.047) in the subgroup with speech worsening*.

Regarding the perceptual rating of speech performance in StimOFF, no significant differences were seen concerning voice, articulation, and prosody, but there was a tendency to higher impairment in the “fluency” category and the sum perceptual score as well (see Table 6). Based upon acoustic analysis, the speech pattern in the OFF condition in the subgroup with speech worsening under stimulation was characterized by significant reduction of Pinw% and higher grade of articulatory acceleration/AA. The other measures of speech rate (NSR and PR%) at least showed a tendency to higher average articulatory velocity/NSR and elevated ratio of speech pauses/PR%. No significant differences were found with the remaining measures of speech (see Table 6). Furthermore, higher UPDRS III scores in the OFF condition were correlated to more pronounced worsening of articulation (*r* = −0.655, *p* = 0.039) and overall speech performance (*r* = −0.608, *p* = 0.055) according to the perceptual speech score. Similarly, higher measures of AA showed a correlation to articulatory worsening (*r* = 0.655, *p* = 0.039) and higher values for jitter, shimmer, and nhR were correlated with an elevation of the perceptual sum speech score under stimulation (*r* = 0.733–0.764, *p* = 0.014–0.019 respectively). No such “OFF condition” pattern or similar correlations could be identified for the subgroup of patients who featured no speech worsening under stimulation.

**Table 6:** **Comparison between the PD subgroup with worsening of speech performance with the PD subgroup with unchanged or amelioration of speech performance under stimulation**.

	PD group with worse speech performance under stimulation/(*n* = 8, 6 male)			PD group with unchanged or better speech performance under stimulation/(*n* = 30, 16 male)	Comparison of patients with speech worsening with the group with unchanged/better speech performance, both in the OFF condition
	StimOFF condition Median/1.–3. quartile	StimON condition Median/1.–3. quartile	Comparison OFF vs. ON	StimOFF condition Median/1.–3. quartile	
UPDRS III	48/40.25–52.75	23.5/13.25–33	*p* < 0.001	47/31.5–46.25	*p* = 0.024
UPDRS speech item	1/1–2	1.5/1–2	n.s.	1/1–2	n.s. (*p* = 0.102)
Perceptual analysis/sum score	6/4.5–7.75	7/7–8.75	*p* = 0.001	5/3–7	n.s. (*p* = 0.122)
Voice	1.5/1–2	1.5/1–2	n.s.	1/1–2	n.s.
Articulation	2/1–2	2/2–2	*p* = 0.033	2/1–2	n.s.
Fluency	2/1–2.75	2.5/1.25–3	n.s. (*p* = 0.08)	1/1–2	n.s. (*p* = 0.065)
Prosody	1/1–2	1.5/1–2	n.s. (*p* = 0.08)	1/0–1	n.s.

**Acoustic analysis**	**Mean/SD**	**Mean/SD**		**Mean/SD**	

Jitter	1.535/1.244	1.560/1.304	n.s.	2.211/2.087	n.s.
Shimmer	8.551/6.256	9.062/7.097	n.s.	11.335/7.483	n.s.
nhR	0.064/0.063	0.061/0.068	n.s.	0.092/0.105	n.s.
Loudness (dB)	71.17/6.70	72.15/4.73	n.s.	69.72/7.83	n.s.
MeanF0 male	124.13/16.90	123.44/15.17	n.s.	117.75/20.09	n.s.
MeanF0 female	168.34/8.05	192.50/0.30	(n.a.)	169.41/45.69	(n.a.)
VAI male	0.681/0.075	0.648/0.065	n.s.	0.648/0.070	n.s.
VAI female	0.704/0.057	0.674/0.154	(n.a.)	0.723/0.063	(n.a.)
Pinw%	8.14/7.72	7.09/7.86	n.s.	17.50/10.56	*p* = 0.025
NSR	5.46/1.48	5.48/1.61	n.s.	5.26/0.99	n.s.
PR%	19.30/10.07	21.26/7.19	n.s.	16.75/8.87	n.s.
AA	0.95/0.73	0.61/0.63	n.s.	0.19/0.40	*p* = 0.022
F_0_SD male	15.94/2.80	14.24/3.93	n.s.	14.30/4.18	n.s.
F_0_SD female	16.16/1.58	19.60/5.99	(n.a.)	16.85/5.68	(n.a.)

*(n.a.): statistical analysis not applicable because of small sample size*.

## Discussion

In the groupwise comparison of speech in the StimOFF and ON conditions, only perceptual assessment of voice quality showed a significant amelioration under STN-DBS, which was mirrored by similar trends toward lower values for the accordant acoustic measures (jitter, shimmer, and nhR) as well as higher values for loudness of speech in the acoustic analysis, however, without statistical significance. These findings are in line with previous investigations reporting on a stimulation-induced improvement of voice quality and loudness, however, not necessarily accompanied by an amelioration of overall speech performance (6–12) that can in general be confirmed by our data. In the current study, perceptual and acoustic measures of articulation, fluency, and prosody showed no consistent behavior under STN-DBS, instead, there was a group of *n* = 8 patients with worsening of overall speech performance that could not be restricted to a consistent pattern but was induced by different degrees of deterioration of articulation, fluency, and prosody.

One main result of the present investigation was the identification of a subgroup with preexisting speech abnormality in the OFF condition that showed a further deterioration under stimulation. The preexisting pattern of dysarthria was found to be characterized by a high degree of articulatory slurring (as mirrored by reduced Pinw%) accompanied by an acceleration of speech in the course of the performance (indicated by significantly elevated AA). Furthermore, these patients featured not only higher overall UPDRS III scores in the OFF condition, but worse global speech performance (according to UPDRS speech item and the perceptual sum score) as well, however without statistical significance which might be due to the small sample size of *n* = 8. In this subgroup of patients with speech deterioration, there also was a correlation between higher values for UPDRS III, articulatory acceleration/AA and poor voice quality (indicated by higher values for jitter, shimmer, and nhR) in the OFF condition and the perceptually detected degree of speech worsening under stimulation.

There are only very few previous studies focused on the preexisting patients’ characteristics which might be “risk factors” for stimulation-induced speech deterioration. Dromey and Bjarnason tested six PD patients with speech deterioration under STN-DBS according to perceptual ratings, however, acoustic measures of articulation and phonation deriving from analysis of speech and non-speech utterances showed mixed results with some speakers improving and others becoming worse on individual measures (27). In another investigation, negative effects on speech intelligibility were found in two out of seven PD patients and were attributed to slight stimulation-induced facial dyskinesia, which was not observed in our study (28). Pützer and coworkers obtained objective measures of phonatory and articulatory movements based upon acoustic analysis of non-speech syllable production in nine PD patients and reported mixed results under stimulation: Precision of glottal and supraglottal articulation as well as the phonatory function was reduced in some speakers, whereas for others an improvement was observed (15). In a subgroup of patients, the accuracy of stop consonant articulation was found to be impaired under stimulation, which shows some relation to our finding of increased Pinw% as a measure of overall articulatory slurring. However, in this previous study, the mixed response of patients’ articulatory capacity had not been related to the speech performance in the StimOFF condition.

Although the present study gives some first indication for specific patient-related risk factors for speech worsening under STN-DBS, there are some undisputable limitations, especially because of the small sample size, which lessens the value of the statistic analysis. Since there was an overlap of values for AA and Pinw% in the “deteriorating” group and the group of patients with no stimulation-induced worsening of speech, positive and negative predictive values for these measures were only poor. Besides, it has to be mentioned that the speech evaluation was based upon a German text and therefore, some of the findings could be language-dependent.

Furthermore, the impact of surgical factors has not been accounted for although in previous studies, the position of the stimulation electrode within the medial and/or posterior portion of the STN was linked with poorer speech intelligibility (19, 29, 30). However, even with electrodes exactly located within the STN, a subgroup of 36% patients was found to feature a deterioration of speech under stimulation in another study (31). This might at least be explained by the explicit stimulation settings in the individual patient since high amplitude and/or high frequency stimulation was consistently found to be a risk factor for worsening of speech (19, 29–34). Furthermore, selective or predominant stimulation of the left-side STN was reported to induce profoundly negative effects on prosody, articulation, and hence, intelligibility (33, 34). In contrast, in our study, the majority of patients with speech deterioration had higher stimulation amplitudes on the right-side STN, and no clear differences were seen concerning frequencies, pulse width and/or the electrode contacts chosen for stimulation. These preliminary observations seem to underline the assumption that surgical or stimulation factors alone cannot account for overall speech performance under STN-DBS.

Another methodical weakness of our investigation is the lack of pre-surgical speech data that would be necessary to rule out a possible microlesion effect of electrode placement. Up till now, there are only very few investigations with speech testings before and at certain follow-up intervals after DBS surgery (11, 19, 28, 35). In the largest of these studies, there was a correlation between poorer speech outcome after 1 year and higher pre-surgical general motor impairment (19). Another study on seven patients found no consistent effects of DBS surgery alone (that is, no hint of the microlesion effect) and no consistent stimulation effect on speech under STN-DBS after 3 months, but a slight improvement of pitch variability and sound pressure levels under stimulation 6 months post-op (27).

Summarized, despite some methodical limitations, the current study provides first evidence for a specific patient-related “risk profile,” namely high overall motor and speech impairment according to UPDRS III and preexisting articulatory slurring and articulatory hastening, which seems to be associated with further decline of speech performance under STN-DBS with stimulation settings optimized for motor function. In this subgroup of patients, a positive effect of STN-DBS on phonatory and voice parameters seems to be outweighed by a pro-dysarthrogenic stimulation effect that is correlated to the degree of AA and overall voice impairment in the OFF condition. Subsequent studies are warranted, especially with pre-surgical speech recordings, to further corroborate these preliminary findings to allow neurologists to pre-surgically estimate the individual risk of deterioration of speech under STN-DBS.

## Author Contributions

Dr. Sabine Skodda – study concept and design, acquisition and analysis of data, statistical analysis, and conception and writing of the manuscript; Dr. Wenke Grönheit – acquisition and analysis of data; Dr. Uwe Schlegel – critical revision of the manuscript for important intellectual content; Dr. Martin Südmeyer – analysis and interpretation of data, revision of the manuscript; Dr. Alfons Schnitzler – critical revision of the manuscript for important intellectual content; Dr. Lars Wojtecki – study concept and design, study supervision, and critical revision of the manuscript; All authors have read the manuscript, and the paper has not previously been published and is not under simultaneous consideration by another journal. There has been no ghost writing by anyone not named on the authors list.

## Conflict of Interest Statement

The authors declare that the research was conducted in the absence of any commercial or financial relationships that could be construed as a potential conflict of interest.
